# Associations Between Right Ventricular Remodeling, Exercise and Circulating Metabolites in Volume and Pressure Overload States

**DOI:** 10.1016/j.jacasi.2025.09.010

**Published:** 2025-10-29

**Authors:** Xiaodan Zhao, Shuang Leng, Ru-San Tan, Fei Gao, Lynette Teo, Ping Chai, Hai Ning Wee, Kee Voon Chua, Jianhong Ching, Sharon Han, Phong Teck Lee, James W. Yip, Ju Le Tan, Jean-Paul Kovalik, Angela S. Koh, Liang Zhong

**Affiliations:** a National Heart Research Institute Singapore, National Heart Centre Singapore, Singapore; b Duke-NUS Medical School, Singapore; c National University Hospital Singapore, Singapore; d Yong Loo Lin School of Medicine, National University of Singapore, Singapore; e Singapore General Hospital, Singapore; f KK Women’s and Children’s Hospital, Singapore; g Department of Biomedical Engineering, National University of Singapore, Singapore

**Keywords:** metabolomics, pressure overload, pulmonary arterial hypertension, repaired tetralogy of Fallot, volume overload

## Abstract

**Background:**

Ventricular pressure and volume overload may induce alterations in myocardial function, exercise capacity, and tissue metabolic pathways.

**Objectives:**

The authors investigated and compared these changes in pulmonary arterial hypertension (PAH) vs repaired tetralogy of Fallot (rTOF) with residual pulmonary regurgitation, which exemplify pressure- and volume-overloaded states, respectively.

**Methods:**

A total of 227 subjects (103 healthy control subjects, 67 rTOF, 57 PAH) identified from an ongoing prospective cohort study all underwent cardiac magnetic resonance (CMR), cardiopulmonary exercise tests (CPET), and serum assays for metabolomic profiling. Measurements included left ventricular end-diastolic volume (LVEDV), right ventricular (RV) end-diastolic volume (RVEDV), right ventricular ejection fraction (RVEF), feature-tracked tricuspid annular plane systolic excursion (TAPSE), and right ventricular global longitudinal strain (RVGLS) on CMR, and peak oxygen uptake (PVo_2_) on CPET.

**Results:**

rTOF had significantly higher RV mass index, RVEDV index, RVEDV/LVEDV, and RV/LV mass ratios vs PAH and control subjects. Compared with control subjects, RV functional parameters (RVEF, TAPSE, and RVGLS) were impaired in both rTOF and PAH. Significant decrements in PVo_2_ were observed from control subjects to rTOF to PAH. Valine, leucine, and isoleucine levels were elevated in rTOF (but not in PAH), which associated negatively with TAPSE and RVGLS. Choline levels and PVo_2_ were associated negatively and positively in rTOF and control subjects, respectively. In PAH, dimethylglycine—which was elevated vs rTOF and control subjects—was associated positively with RVEDV/LVEDV and RV/LV mass, and negatively with RVEF, TAPSE, and RVGLS.

**Conclusions:**

PAH and rTOF exhibited distinct RV remodeling, and PAH, worse exercise tolerance. Differences in circulating branched chain amino acids and one-carbon metabolites were observed among rTOF, PAH, and control subjects.

Hemodynamic load—preload and afterload during ventricular diastolic filling and systolic contraction, respectively—is a critical regulator of myocardial function, gene expression, and phenotype.^1^ Histologically, increased afterload (“pressure overload”) induces maladaptive left ventricular (LV) hypertrophy with increased fibrosis, inflammation, and cardiomyocyte apoptosis. By contrast, increased preload (“volume overload”) leads to LV hypertrophy without increased fibrosis and inflammation, and with comparatively less apoptosis.^1^ Analogous to the LV, the right ventricle (RV) undergoes hypertrophy and dilatation, with extensive fibrosis, when exposed to RV pressure overload, and dilatation with mild fibrosis, when volume-overloaded.^2^ On echocardiography, systolic and diastolic flattening of the interventricular septum qualitatively distinguish between severe RV pressure- vs volume-overloaded states, respectively.^2^ Quantitatively, speckle-tracked RV global longitudinal strain (GLS) and RV free wall strain^3^ and 3-dimensional echocardiography–derived ventricular-arterial coupling ratio (RV stroke volume/RV end-systolic volume)^4^ and RV regional curvature, longitudinal, and radial functions^5^ were more pronounced in the pressure- vs volume-overloaded patient groups. Cardiac magnetic resonance (CMR), recognized as the gold standard for the quantification of cardiac chamber volumes and function due to its excellent accuracy and reproducibility,^6^ is less often studied. In pressure-overloaded pulmonary arterial hypertension (PAH) vs volume-overloaded repaired tetralogy of Fallot (rTOF) patients, CMR feature-tracked GLS was compared and reported on their prognostic utility.^7^ Separate studies were published on CMR measurements (including 4-dimensional [4D] CMR flow-derived energetics) in conjunction with objective assessments of exercise capacity by cardiopulmonary exercise test (CPET) in PAH^8^ and rTOF.^9^

In normal adult heart, fatty acid oxidation is the major contributor of adenosine-triphosphate production, around 60% to 90%, and the remainder is generated by glucose metabolism.^2^ With comorbidities, there might be a shift in the predominant metabolic pathway in the heart with less dependency on fatty acid oxidation pathway. In pressure-overloaded PAH, the RV wall becomes hypertrophied with higher afterload caused by increased pulmonary vascular resistance. At the cellular level, ventricular pressure overload leads to significant alterations in myocardial proteins involved in pathways related to mitochondrial function and oxidative stress that are crucial for maintaining chamber structure and function under pressure. Serum metabolomic profiling is increasingly being used to investigate tissue metabolic pathways in combination with cardiovascular imaging.^10^ For RV pressure overload in humans, distinct serum metabolomics profiles, including polyamine, histidine, and sphingomyelin pathways, are seen that associate with RV dilation on transthoracic echocardiogram, measures of disease severity in PAH, and mortality.^11^ Compared with pressure overload, RV volume overload at the cellular level is less extensively studied, and whether it exhibits similar or different metabolomic signatures has not been elucidated. This underscores a significant gap in our current understanding of molecular mechanisms that underpin RV dysfunction in rTOF patients.

To our knowledge, a direct and detailed comparison of RV pressure- vs volume-overloaded clinical models integrating CMR and exercise phenotyping, as well as incorporating molecular signal annotation, is currently lacking. In a carefully characterized prospective cohort of patients, we compared clinical characteristics, extent of RV function and remodeling, exercise capacity, and circulating metabolites in PAH vs rTOF, which exemplify RV pressure- and volume-overloaded clinical states, respectively.

## Methods

### Study population

The study population was derived from the INITIATE registry (Integrated Computational modelIng of Right Heart Mechanics and Blood Flow Dynamics in Congenital Heart Disease; NCT03217240), which recruited PAH ad rTOF patients, as well as control subjects free of cardiovascular diseases between June 2017 and February 2022.^8^^,^^9^^,^^12^^,^^13^ The criteria for inclusion and exclusion are given in the Supplemental Methods. For this analysis, we selected adult subjects with complete CMR, CPET, and metabolomics profiling, resulting in a final sample of 103 control subjects, 67 rTOF and 57 PAH. Part of the study population have been used in our previous publications investigating range values of 4D flow CMR in healthy control subjects,^8^^,^^9^^,^^12^, ^13^, ^14^ rTOF^9^^,^^14^ and PAH.^8^ This study had been approved by the institutional review board, and written informed consent was obtained from each subject.

### Data acquisition

#### CMR protocol

CMR acquisition was performed on Ingenia 3.0T (Philips Healthcare) and Magnetom Aera 1.5T (Siemens Healthineers) scanners, as previously published.^8^^,^^9^^,^^12^^,^^13^ Balanced steady-state free precession end-expiratory breath-hold cine images were acquired in multiplanar long-axis views (2-, 3-, and 4-chamber) and a stack of parallel short-axis views covering the entire LV and RV. These images were then reconstructed with a temporal resolution of 30 frames per cardiac cycle. Standard through-plane 2-dimensional phase-contrast pulmonary artery (PA) flow imaging was acquired just above the pulmonary valve in a plane perpendicular to the long-axis pulmonary trunk view. The standard cine image parameters for both scanners are provided in Supplemental Table 1.

#### Blood sample collection

Nonfasting, antecubital venous blood samples (20-30 mL) were collected on the day of CMR acquisition. After collection, the blood samples were immediately placed on ice for transportation and were processed within 6 hours to obtain serum samples, which were subsequently stored at −80 °C.

#### Cardiopulmonary exercise testing

All subjects underwent CPET at a central laboratory within 1 week after CMR imaging. Peak oxygen uptake (PVo_2_), % predicted PVo_2_, minute ventilation (VE), oxygen consumption, and carbon dioxide output (VCo_2_) were calculated using standard protocols. The detailed protocols are provided in the Supplemental Methods. Patients were stratified into abnormal exercise capacity group if PVo_2_ ≤15 mL/kg/min, or % predicted PVo_2_ ≤65% or VE/VCo_2_ slope ≥36.^15^

### Data Analysis

#### Biventricular Measurements

A fully automated artificial intelligence–based approach was used to segment endocardial and epicardial borders in stacks of LV and RV short-axis images at end-diastole and end-systole.^8^^,^^9^^,^^13^^,^^16^ Manual correction was performed where needed after visually inspecting the segmentation contours. Papillary and trabecular muscles were included in the volume calculation. LV mass was measured at end-diastole. LV mass as well as all LV and RV volumetric parameters—end-diastolic volume, end-systolic volume, stroke volume (SV)—were indexed to body surface area. Pulmonary regurgitant fraction was calculated from PA flow curve by semiautomatically tracing the PA contours from the 2-dimensional phase-contrast flow images through the cardiac cycle.

#### Tricuspid Annular Velocities, Displacement, and RVGLS

Our CMR-based feature tracking software was used to derive the tricuspid annular velocities during systole (S′), early diastole (E′), late diastole (A′), tricuspid annular plane systolic excursion (TAPSE)—tricuspid annular displacement at end-systole and RV global longitudinal strain (GLS). This software can semiautomatically track the medial and lateral tricuspid valve insertions, and RV epicardial apex in the 4-chamber view. The excellent reproducibility and applications of these parameters have been reported in our previous publications.^7^^,^^17^

#### Metabolomics Profiling

In fatty acid oxidation, acylcarnitines are crucial to transport long-chain fatty acids into mitochondria for β-oxidation. The levels of specific acylcarnitines—accumulation or excretion—can be indicative of defects in fatty acid oxidation, leading to metabolic disorders, which can be identified through metabolic profiling in the blood. Branched-chain amino acids (BCAAs) refer to 3 essential amino acids—leucine, isoleucine, and valine—and they play a crucial role in muscle growth, recovery, and overall health. The link between BCAAs and a variety of cardiovascular diseases is well-established, for example, heart failure (HF) and coronary artery disease,^18^ while in rTOF and PAH, and comparison of both disease states have not been investigated. One-carbon metabolism (OCM) is a vital pathway to transfer one-carbon units, and its dysregulation could disrupt cardiac cell development yielding structural abnormalities. In the current study, we performed the serum metabolomic profiling analysis in the Duke-NUS Metabolomics Facility as previously described,^19^, ^20^, ^21^ and the detailed procedures for acylcarnitine and nitrogen-containing compounds analysis are provided in the Supplemental Methods. In total, 131 metabolites were analyzed, comprising 69 acylcarnitine, 21 amino acid, and 41 nitrogen pathway metabolites (Supplemental Table 2).

### Statistical Analysis

Continuous variables are presented as median (25th-75th percentiles), and categorical data as n (%). For continuous variables, comparison of 2 groups was performed using Student’s *t*-test for normally distributed data and Mann-Whitney *U* test for non-normally distributed data. Chi-square test was used for categorical data. Comparison of means for more than 2 groups was performed using nonparametric 1-way analysis of variance with post hoc pairwise comparisons in event of a significant Kruskal-Wallis test. Only raw *P* values were reported, and it is left to the reader to apply any desired significance level calculation. Association between 2 variables was assessed using Pearson’s correlation coefficient. To identify metabolites correlations and reduce the dimensionality of correlated metabolites, we performed sparse principal component analysis (SPCA), which used a penalized matrix decomposition.^22^ Compared with regular principal component analysis, which suffers from the fact of a dense loading matrix from all variables, SPCA is capable of producing sparse loadings that makes it more biologically interpretable. The missing data were inputted with 0.01. Before conducting SPCA, mean centering was performed by subtracting the mean of each variable from its corresponding data point to ensure that the analysis is performed around the origin of the transformed coordinate system. After this, we normalized the distributions of all metabolites using a logarithmic transformation. Specifically, we set the orthogonality constraint^22^ on each component and the number of components to be 10, and reported each component’s description and variance-accounted proportion. To facilitate factor comparisons across 3 groups, 2 general linear models were constructed: “Basic” was adjusted for age and sex; and “Fully adjusted” was adjusted for age, sex, body surface area, systolic blood pressure, diastolic blood pressure, heart rate, diabetes, hyperlipidemia, hypertension, and current smoker status. Significant metabolite factors were then compared pairwise between groups if both original and adjusted *P* values from the fully adjusted analysis of covariance (ANCOVA) and Benjamini-Hochberg procedure, respectively, are <0.05. Receiver-operating characteristic (ROC) analysis was performed to investigate the discriminative utility of individual metabolites to predict exercise intolerance in patient subgroups, and DeLong’s test was used to compare the significance of area under the ROC curve (AUC) values. *P* < 0.05 was considered statistically significant.

## Results

### Demographics and Clinical Characteristics

A total of 103 control subjects (median age 46 years, 39 women), 67 rTOF (median age 31 years, 40 women), and 57 PAH (median age 46 years, 49 women) were analyzed, and all of them completed metabolomics profiling. The baseline demographic and clinical characteristics of the three groups are presented in Table 1. PAH group had higher percentage of functional class World Health Organization (WHO) functional class >I than rTOF group (38.6% [22/57] vs 17.9% [12/67]). In PAH cohort, the median values of NT-proBNP (N-terminal pro–B-type natriuretic peptide), right atrial pressure, mean pulmonary arterial pressure, pulmonary capillary wedge pressure, pulmonary vascular resistance, and RV systolic pressure were 256.6 pg/mL, 8.0 mm Hg, 47.0 mm Hg, 10.0 mm Hg, 9.5 WU, and 72.0 mm Hg, respectively. Most of the PAH patients were on endothelin receptor antagonists (n = 32/57, 56.1%) and phosphodiesterase inhibitors (n = 47/57, 82.5%).

**Table 1:** Demographic Characteristics, CMR Function, and CPET Parameters of Study Participants

	Control Subjects (n = 103)	rTOF (n = 67)	PAH (n = 57)	*P* Value			
				Overall	Control Subjects vs rTOF	Control Subjects vs PAH	rTOF vs PAH
Demographics							
Age, y	46 (30-59)	31 (26-41)	46 (37-59)	<0.001	<0.001	0.247	<0.001
Male/female, n	64/39	27/40	8/49	<0.001	0.007	<0.001	0.001
Height, cm	167 (159-172)	163 (157-171)	158 (153-164)	<0.001	0.060	<0.001	0.003
Weight, kg	64.1 (55.6-72.2)	61.5 (53.2-75.3)	58.4 (52.3-67.5)	0.056	0.472	0.012	0.176
Heart rate, beats/min	71 (61-80)	75 (69-79)	79 (71-92)	<0.001	0.020	<0.001	0.066
NYHA Functional class >WHO I	—	12 (17.9)	22 (38.6)	—	—	—	0.015
Clinical features							
Body surface area, m^2^	1.71 (1.59-1.83)	1.66 (1.52-1.88)	1.60 (1.46-1.73)	0.003	0.225	<0.001	0.067
Body mass index, kg/m^2^	22.8 (20.7-25.1)	23.5 (19.8-26.9)	23.4 (20.6-26.4)	0.762	0.503	0.614	0.812
SBP, mm Hg	131 (120-140)	119 (112-135)	115 (104-130)	<0.001	0.001	<0.001	0.110
DBP, mm Hg	79 (71-87)	72 (66-79)	68 (63-78)	<0.001	0.001	<0.001	0.144
LV function							
LV mass index, g/m^2^	45 (38-50)	43 (36-54)	50 (42-57)	0.014	0.674	0.005	0.021
LVEDV index, mL/m^2^	73 (65-82)	69 (60-78)	62 (50-69)	<0.001	0.035	<0.001	<0.001
LVESV index, mL/m^2^	29 (24-34)	28 (21-34)	18 (12-25)	<0.001	0.193	<0.001	<0.001
LVSV index, mL/m^2^	44 (39-48)	40 (36-47)	42 (35-47)	0.018	0.012	0.031	0.964
LV ejection fraction, %	60 (57-63)	59 (54-66)	70 (62-79)	<0.001	0.522	<0.001	<0.001
RV function							
RV mass index, g/m^2^	16 (14-18)	27 (24-32)	24 (20-30)	<0.001	<0.001	<0.001	0.010
RVEDV index, mL/m^2^	78 (68-91)	133 (116-152)	89 (72-110)	<0.001	<0.001	0.002	<0.001
RVESV index, mL/m^2^	37 (29-41)	68 (58-78)	45 (31-74)	<0.001	<0.001	0.001	<0.001
RVSV index, mL/m^2^	43 (38-48)	62 (56-72)	43 (37-50)	<0.001	<0.001	0.903	<0.001
RV ejection fraction, %	54 (50-59)	48 (44 52)	47 (36-60)	<0.001	<0.001	0.002	0.996
RVEDV/LVEDV	1.07 (1.01-1.14)	1.97 (1.69-2.28)	1.45 (1.24-2.04)	<0.001	<0.001	<0.001	<0.001
RV/LV mass	0.37 (0.33-0.42)	0.63 (0.53-0.75)	0.49 (0.38-0.66)	<0.001	<0.001	<0.001	<0.001
RV mass/RVEDV, g/mL	0.21 (0.19-0.22)	0.21 (0.18-0.24)	0.25 (0.23-0.29)	<0.001	0.782	<0.001	<0.001
Pulmonary regurgitation fraction, %	0.94 (0.32-1.82)	44.6 (33.5-54.8)	2.24 (0.65-7.83)	<0.001	<0.001	<0.001	<0.001
CMR feature tracking							
S′, cm/s	10.1 (9.1-11.3)	7.8 (6.2-9.0)	8.1 (6.7-9.4)	<0.001	<0.001	<0.001	0.186
E′, cm/s	11.6 (9.2-14.0)	10.2 (8.1-12.1)	7.3 (5.6-8.8)	<0.001	0.006	<0.001	<0.001
A′, cm/s	10.2 (9.1-11.5)	6.6 (4.7-7.9)	10.5 (7.1-12.4)	<0.001	<0.001	0.693	<0.001
E′/A′	1.12 (0.85-1.43)	1.69 (1.19-2.17)	0.68 (0.50-1.09)	<0.001	<0.001	<0.001	<0.001
TAPSE, mm	20.4 (18.1-22.4)	14.3 (12.7-16.9)	14.8 (13.0-17.8)	<0.001	<0.001	<0.001	0.724
RVGLS, %	23.7 (21.4-26.2)	18.8 (17.2-22.0)	17.3 (13.8-21.2)	<0.001	<0.001	<0.001	0.027
CPET parameters							
PVo_2_, mL/kg/min	23.0 (18.8-28.9)	17.9 (14.2-21.5)	11.8 (10.4-14.0)	<0.001	<0.001	<0.001	<0.001
METs	6.6 (5.4-8.3)	5.0 (4.1-6.1)	3.4 (3.0-4.0)	<0.001	<0.001	<0.001	<0.001
% Predicted PVo_2_, %	89 (74-106)	67 (58-78)	52 (42-64)	<0.001	<0.001	<0.001	<0.001
VE/VCo_2_ slope	26 (24-28)	27 (25-30)	40 (37-43)	<0.001	0.035	<0.001	<0.001

Values are median (25th-75th percentile) or n (%), unless otherwise indicated.

The Fisher exact test was used for categorical variables. The Kruskal-Wallis test was used for continuous variables with 3 groups. The Mann-Whitney *U* test was used for continuous variables with 2 groups.

A′ = tricuspid annular peak late diastolic velocity; CMR = cardiovascular magnetic resonance; CPET = cardiopulmonary exercise test; DBP = diastolic blood pressure; E′ = tricuspid annular peak early diastolic velocity; LV = left ventricular; LVEDV = left ventricular end-diastolic volume; LVESV = left ventricular end-systolic volume; LVSV = left ventricular stroke volume; METs = metabolic equivalents; PAH = pulmonary arterial hypertension; PVo_2_ = peak oxygen uptake; rTOF = repaired tetralogy of Fallot; RV = right ventricular; RVEDV = right ventricular end-diastolic volume; RVESV = right ventricular end-systolic volume; RVGLS = right ventricular global longitudinal strain; RVSV = right ventricular stroke volume; S′ = tricuspid annular peak systolic velocity; SBP = systolic blood pressure; TAPSE = tricuspid annular peak systolic excursion; VCo_2_ = carbon dioxide production; VE = minute ventilation; WHO = World Health Organization.

Compared with control subjects, both patient groups had more females, higher heart rate, and smaller systolic and diastolic blood pressure (Table 1). Both patient groups had significantly reduced left ventricular end-diastolic volume (LVEDV) index, LV SV index, right ventricular ejection fraction (RVEF), S′, E′, TAPSE, and right ventricular global longitudinal strain (RVGLS) compared with control subjects. rTOF and PAH patients had increased RV mass index, right ventricular end-diastolic volume (RVEDV) index, right ventricular end-systolic volume (RVESV) index, RVEDV/LVEDV, and RV/LV mass compared with control subjects. rTOF and PAH had similar percentages of diabetes, hypertension, and hyperlipidemia, and PAH had a higher percentage of WHO functional class >I. When comparing between rTOF and PAH, we noted that PAH had significantly larger LV mass index, LV ejection fraction, and RV mass/RVEDV, and smaller LVEDV index and LVESV index compared with rTOF (all *P* < 0.05) (Table 1). rTOF had significantly increased RV mass index, RVEDV index, RVESV index, RVSV index, RVEDV/LVEDV, RV/LV mass, and pulmonary regurgitant fraction compared with PAH. Both S′ and TAPSE were significantly increased in control subjects, but comparable between rTOF and PAH. From control subjects to rTOF to PAH, there was a significant decrease for E′, RVGLS, PVo_2_, METs, % predicted PVo_2_, and increasing trend in VE/VCo_2_ slope (all *P* < 0.05) (Table 1).

### Comparison of SPCA Metabolite Factors and Individual Metabolites

Ten SPCA-derived metabolite factors were identified, clustering in biologically related groupings with detailed principal component analysis results given in Supplemental Table 3. In the basic model, omnibus ANCOVA identified 6 metabolite factors (X1 to X6) significant at the Bonferroni-corrected significance threshold (*P* < 0.0001) (Table 2). Five metabolite factors were all significant in the full model (*P* ≤ 0.001), whereas factor X3 was no longer significant (*P* = 0.011). Only factors X5 and X6 showed significant differences for each pairwise comparison from the fully adjusted model, with the least square means of PAH, rTOF, and control subjects being 3.20, 1.33, and 0.091 for factor X5, and −1.71, −0.69, and 0.57 for factor X6, respectively (Table 2).

**Table 2:** Metabolite Factor Means and Comparisons Between rTOF, PAH, and Control Subjects

Factor	Description	ANCOVA			Pairwise Comparisons From Full Model			Metabolite Factor Mean Values^a^		
		Basic^b^	Fully Adjusted^c^	BHP Adjusted^d^	rTOF vs Control Subjects	PAH vs Control Subjects	PAH vs rTOF	PAH (n = 57)	rTOF (n = 67)	Control Subjects (n = 103)
1	Acylcarnitines	<0.0001	0.001	0.002	0.060	0.001	0.32	−4.13 (1.12)	−2.84 (1.07)	−1.18 (1.21)
2	Amino acid–related	<0.0001	<0.0001	<0.001	<0.0001	0.068	0.13	−1.07 (0.94)	−2.44 (0.91)	0.49 (1.03)
3	Acylcarnitines and methylamine-related	<0.0001	0.011	0.018	0.026	0.039	1.0	1.47 (0.68)	1.40 (0.66)	0.25 (0.74)
4	Short- and medium chain acylcarnitines, amino acids and nitrogen transfer	<0.0001	<0.0001	<0.001	<0.0001	<0.0001	0.62	2.56 (0.56)	2.05 (0.54)	−1.25 (0.61)
5	Acylcarnitines and nitrogen transfer	<0.0001	<0.0001	<0.001	0.027	<0.0001	0.001	3.20 (0.74)	1.33 (0.71)	0.091 (0.80)
6	Short- and long-chain acylcarnitines and nitrogen- and one-carbon transfer	<0.0001	<0.0001	<0.001	0.001	<0.0001	0.021	−1.71 (0.52)	−0.69 (0.50)	0.57 (0.57)
7	Acylcarnitines and nitrogen transfer	0.082	0.29	0.322				1.31 (0.57)	0.67 (0.55)	0.86 (0.62)
8	Short-chain acylcarnitines and amino acids and one-carbon transfer	0.14	0.053	0.066				0.86 (0.46)	0.41 (0.45)	1.12 (0.50)
9	Acylcarnitines and nitrogen Transfer	0.93	0.53	0.53				0.81 (0.43)	0.54 (0.41)	0.46 (0.47)
10	Acylcarnitines and nitrogen transfer	0.052	0.019	0.027	1.000	0.065	0.022	0.025 (0.44)	−0.82 (0.42)	−0.70 (0.48)

ANCOVA = analysis of covariance; other abbreviations as in Table 1.

a Data are represented as least square means, adjusted for 10 covariates (age, sex, body surface area, systolic and diastolic blood pressure, heart rate diabetes, hyperlipidemia, hypertension and current smoker). SEM is provided beneath each value.

b *P* values for basic model, adjusted for age and sex.

c *P* values for full model, adjusted for 10 covariates (age, sex, body surface area, systolic and diastolic blood pressure, heart rate, diabetes, hyperlipidemia, hypertension, and current smoker).

d Adjusted *P* value for the fully adjusted model using Benjamini-Hochberg procedure (BHP).

**Figure 1:**
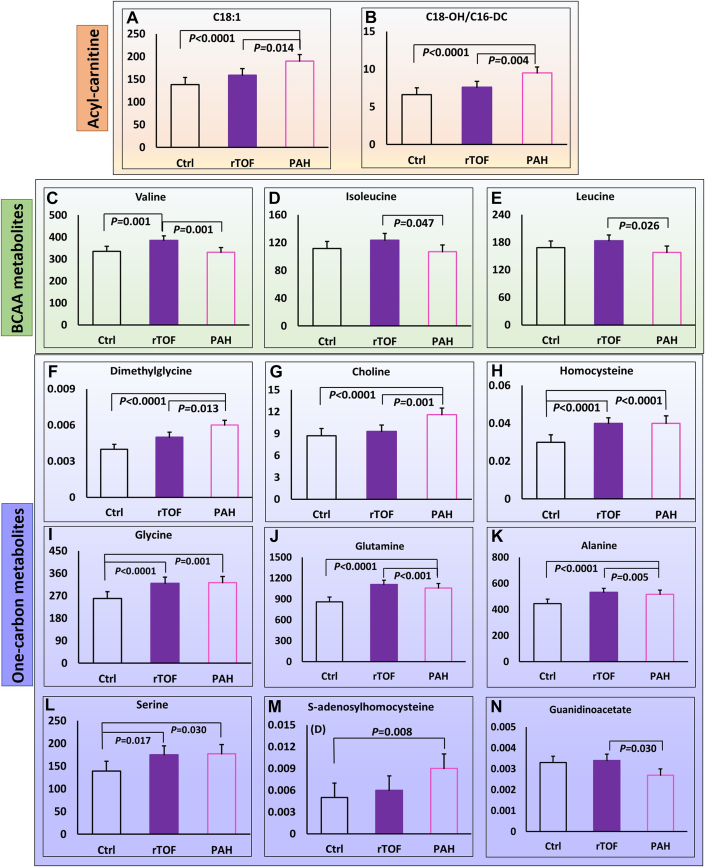
Boxplot Comparisons of Metabolites Among Control, rTOF, and PAH Groups Individual metabolites were compared among 3 groups (control [Ctrl], repaired tetralogy of Fallot [rTOF] and pulmonary arterial hypertension [PAH]) in terms of acylcarnitine (top, A and B), branched-chain amino acid (BCAA) metabolites (middle, C to E); and one-carbon metabolites (bottom, F to N). *P* values are from the pairwise companion from full model, adjusted for age, sex, body surface area, systolic and diastolic blood pressure, heart rate, diabetes, hyperlipidemia, hypertension, and current smoker.

From Supplemental Table 3, we selected important individual metabolites and compared them among the 3 groups using both omnibus ANCOVA and the fully adjusted model (Supplemental Table 4). The heat map plot for these metabolites was given in Supplemental Figure 1 after normalizing, standardizing by the *z*-score and averaging with ranges from −1 to 1. For both models, statistically significant differences were observed for acylcarnitine metabolites (C16, C18:1, C14, C18-OH/C16-DC, C18:2, C14-OH/C12-DC) with patient groups having higher values than control subjects (Figures 1A and 1B), and BCAAs (valine, isoleucine, and leucine) with rTOF having significant higher values than PAH (Figures 1C to 1E). Among one-carbon metabolites, homocysteine, dimethylglycine, S-adenosylhomocysteine (SAH), glutamine, glycine, alanine, serine, choline, and guanidinoacetate demonstrated significant differences for omnibus ANCOVA and fully adjusted models (Supplemental Table 4). In particular, PAH had significant increases in dimethylglycine and choline compared with control subjects and rTOF (Figures 1F and 1G). Compared with control subjects, homocysteine, glutamine, glycine, alanine, and serine were significantly increased in both rTOF and PAH, with no differences between 2 groups (Figures 1H to 1L). PAH had significant elevation in SAH compared with control subjects (Figure 1M) and decrease in guanidinoacetate compared with rTOF (Figure 1N).

### Different Associations Between Metabolites, CMR Function, and Exercise According to Loading Conditions

**Figure 2:**
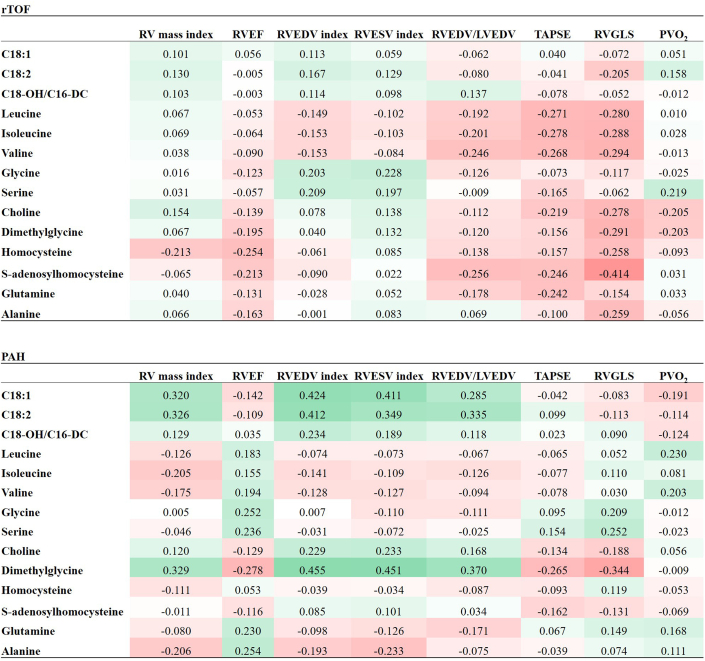
Correlation Coefficients Between CMR, Exercise, and Metabolites rTOF and PAH The relationship between CMR parameter, PVo_2_, and metabolic parameters in rTOF and PAH, respectively. CMR = cardiac magnetic resonance; LVEDV = left ventricular end-diastolic volume; PVo_2_ = peak oxygen uptake; RV = right ventricle; RVEDV = right ventricular end-diastolic volume; RVEF = right ventricular ejection fraction; RVESV = right ventricular end-systolic volume; RVGLS = right ventricular global longitudinal strain; TAPSE = tricuspid annular plane systolic excursion; other abbreviations as in Figure 1.

Figure 2 presents the correlation coefficients of these parameters in rTOF and PAH. Valine and SAH negatively correlated with RVEDV/LVEDV (*r* = −0.25 [95% CI: −0.46 to −0.006]; −0.26 [95% CI: −0.468 to −0.017]; *P* < 0.05). Choline, dimethylglycine, homocysteine, SAH, and alanine were all negatively correlated with RVGLS (*r* = −0.28 [95% CI: −0.485 to −0.040], −0.29 [95% CI: −0.497 to −0.055], −0.26 [95% CI: −0.469 to −0.019], −0.41 [95% CI: −0.595 to −0.192], and −0.26 [95% CI: −0.470 to −0.020]; *P* < 0.05). BCAAs were all negatively related with TAPSE and RVGLS (*P* < 0.05). Only homocysteine was correlated negatively with right ventricular ejection fraction (RVEF) (*r* = −0.25 [95% CI: −0.466 to −0.015]; *P* < 0.05).

In PAH, elevations of C18:1, C18:2, and dimethylglycine were associated with increased RV mass index (*r* = 0.32 [95% CI: 0.065-0.536], 0.33 [95% CI: 0.072-0.541], and 0.33 [95% CI: 0.075-0.543]; *P* < 0.05), RVEDV index (*r* = 0.42 [95% CI: 0.183-0.616], 0.41 [95% CI: 0.169-0.607], and 0.46 [95% CI: 0.220-0.639]; *P* < 0.01), RVESV index (*r* = 0.41 [95% CI: 0.169-0.607], 0.35 [95% CI: 0.097-0.559], and 0.45 [95% CI: 0.216-0.637]; *P* < 0.01), and RVEDV/LVEDV (*r* = 0.29 [95% CI: 0.026-0.508], 0.34 [95% CI: 0.081-0.548], and 0.37 [95% CI: 0.121-0.575]; *P* < 0.05). Dimethylglycine was negatively associated with RVEF (*r* = −0.28 [95% CI: −0.502 to −0.019]; *P* < 0.05), TAPSE (*r* = −0.27 [95% CI: −0.492 to −0.005]; *P* < 0.05), and RVGLS (*r* = −0.34 [95% CI: −0.555 to −0.092]; *P* < 0.01), and positively with RV/LV mass (*r* = 0.27 [95% CI: 0.008-0.494]; *P* < 0.05).

### Associations of Individual Metabolites with Exercise Capacity in Different Loading Conditions

**Figure 3:**
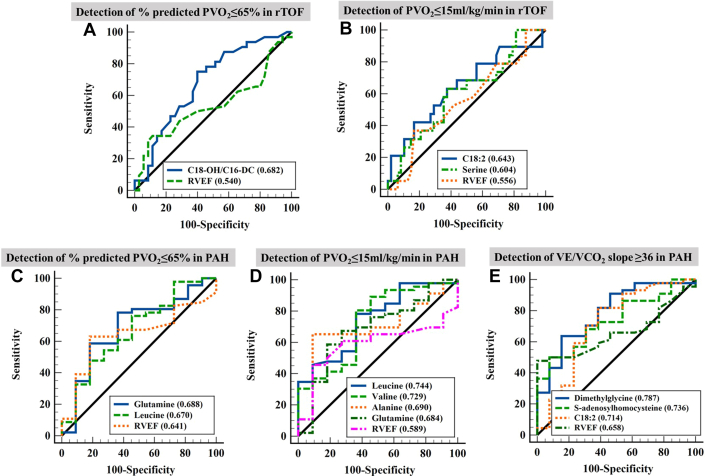
Utility of Metabolites in Detecting Abnormal Exercise Capacity Receiver-operating characteristic analysis was used to study the discrimination utility of individual metabolites for detecting abnormal exercise capacity using different criteria in rTOF and PAH. (A) % predicted PVo_2_ ≤65% in rTOF; (B) PVo_2_ ≤15 mL/kg/min in rTOF; (C) % predicted PVo_2_ ≤65% in PAH; (D) PVo_2_ ≤15 mL/kg/min in PAH; (E) VE/VCo_2_ slope ≥36 in PAH. VE = minute ventilation; VCo_2_ = carbon dioxide production; other abbreviations as in Figures 1 and 2.

In rTOF, ROC analysis demonstrated that C18-OH/C16-DC had higher AUC than RVEF (0.682 [95% CI: 0.552-0.811] vs 0.540 [95% CI: 0.395-0.685]; *P* = 0.165) for detecting abnormal exercise capacity (% predicted PVo_2_ ≤65%, n = 32) (Figure 3A); C18:2 (AUC = 0.643 [95% CI: 0.485-0.800]) and serine (AUC = 0.604 [95% CI: 0.451-0.758]) were noninferior when compared with RVEF (AUC = 0.556 [95% CI: 0.400-0.712]) to detect rTOF with PVo_2_ ≤15 mL/kg/min (n = 19) (Figure 3B) with *P* = 0.429 and *P* = 0.648, respectively. In PAH, ROC analysis showed that glutamine (AUC = 0.688 [95% CI: 0.500-0.875]; *P* = 0.744) and leucine (AUC = 0.670 [95% CI: 0.481-0.859]; *P* = 0.840) were comparable with RVEF (AUC = 0.641 [95% CI: 0.475-0.807]) for detecting abnormal exercise capacity (% predicted PVo_2_ ≤65%, n = 46) (Figure 3C); leucine (AUC = 0.744 [95% CI: 0.584-0.904]; *P* = 0.198), valine (AUC = 0.729 [95% CI: 0.551-0.908]; *P* = 0.277), alanine (AUC = 0.690 [95% CI: 0.514-0.865]), and glutamine (AUC = 0.684 [95% CI: 0.498-0.870], *P* = 0.490) had numerically higher AUCs than RVEF (AUC = 0.589 [95% CI: 0.430-0.747]) for detecting PVo_2_ ≤15 mL/kg/min (n = 46) (Figure 3D) although significance was not reached; dimethylglycine (AUC = 0.787 [95% CI: 0.643-0.931]; *P* = 0.261), SAH (AUC = 0.736 [95% CI: 0.595-0.877]; *P* = 0.456), and C18:2 (AUC = 0.714 [95% CI: 0.529-0.900]; *P* = 0.658) were also better than RVEF (AUC = 0.658 [95% CI: 0.517-0.799]) for detecting VE/VCo_2_ slope ≥36 (n = 44) (Figure 3E).

## Discussion

We present novel observations that characterize differences in RV dysfunction and remodeling, exercise capacity and metabolites between pressure-overloaded PAH and volume-overloaded rTOF by a combination of deep CMR analysis, cardiopulmonary exercise testing, and mass spectrometry–based targeted approaches. The main findings are firstly, although rTOF had more dilated RV than PAH, the exercise capacity was better in this cohort. Secondly, in rTOF, BCAAs (valine, isoleucine, and leucine) were significantly elevated compared with PAH, and higher BCAAs were associated with lower TAPSE and RVGLS. Lastly, in PAH, dimethylglycine was significantly increased compared with control subjects and rTOF, associated positively with RV dilation and RV remodeling, but negatively associated with RV dysfunction (RVEF, TAPSE, and RVGLS) (Central Illustration).

**Central Illustration:**
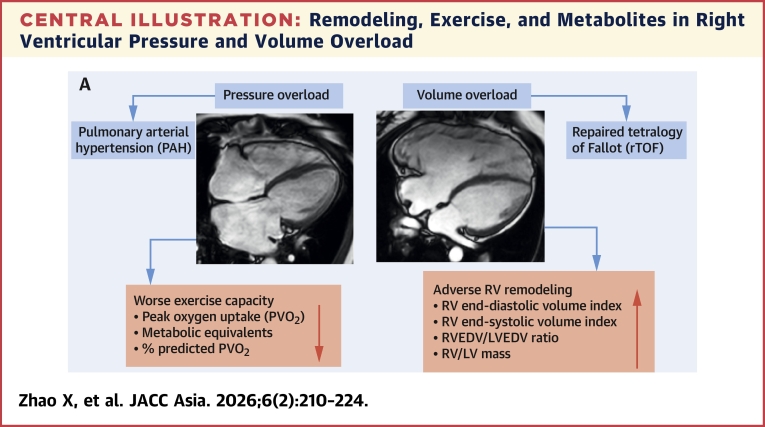

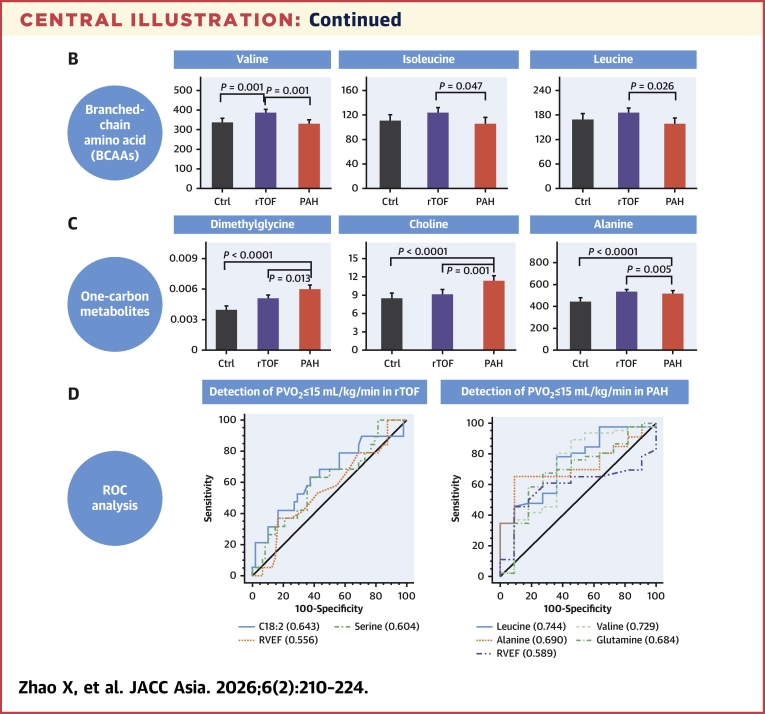
Remodeling, Exercise, and Metabolites in Right Ventricular Pressure and Volume Overload (A) Pulmonary arterial hypertension (PAH) patients with right ventricular (RV) pressure overload have worse exercise capacity in terms of peak oxygen uptake (PVo_2_), metabolic equivalent, and % predicted PVo_2_; repaired tetralogy of Fallot (rTOF) patients with RV volume overload have more adverse RV remodeling in terms of increased right ventricular end-diastolic volume (RVEDV) index, RV end-systolic volume index, RVEDV/left ventricular (LV) end-diastolic volume (LVEDV) ratio, and RV/LV mass. (B) rTOF has significantly elevated levels of branched-chain amino acids (BCAAs) (valine, isoleucine, leucine) than PAH. (C) PAH had significantly higher levels of one-carbon metabolites (dimethylglycine, choline and alanine) than rTOF and healthy control subjects. (D) Receiver-operating characteristic (ROC) analysis demonstrated the better discriminative capacity of individual metabolites than right ventricular ejection fraction (RVEF) in predicting abnormal exercise capacity in terms of PVo_2_.

### RV Function, Remodeling, and Exercise in PAH vs rTOF

Direct comparison of both loading conditions using CMR on the right heart has been explored by Leng et al^7^ using an in-house rapid RVGLS assessment that measures linear distances between the tricuspid annular landmarks and the cardiac apex. Although both loading conditions eventually lead to RV failure or death, disease progression and adaption in RV remodeling from early to end stages are different, as remodeling can occur in response to multiple factors such as loading conditions, myocardial fibrosis, and electromechanical dyssynchrony induced by right bundle branch block.^23^ In PAH, mortality remains unacceptably high among patients at intermediate- and high-risk stages,^24^ and survival has not improved over time despite advancements in therapeutic options.^25^ In pressure-overloaded PAH, the RV adapts to the increased vascular load through increasing muscle contractility and wall thickness during the early stage, also known as the “coupling” stage. As the disease progresses, the hypertrophic process will stop and RV stroke volume will decrease. To maintain SV, RV starts to dilate; and to maintain cardiac output with decreasing SV, the heart rate increases as observed in our sample (Table 1). All of these changes lead to increases in wall stress, oxygen consumption per gram, and arterial elastance (Ea), decrease in ventricular-arterial coupling (Ees/Ea) and leftward septal bowling, and eventually RV uncoupling and failure in the advanced stage with high metabolic demand and reduced output.^26^ Therefore, exercise capacity was significantly reduced as observed in our findings (Table 1).

A common complication following TOF repair is pulmonary regurgitation, which leads to RV dilation, RV dysfunction, and chronic RV volume overload.^27^ We observed that RV adverse remodeling was more severe in rTOF than PAH in terms of RVEDV/LVEDV, although RVEF and TAPSE were comparable. Increased RV mass and reduced RVEF are often related to exercise intolerance in PAH patients, and in our cohort, their correlations with peak oxygen uptake were weak (−0.202 for RV mass and 0.285 for RVEF). Among rTOF patient, such associations were even weaker. This shortcoming underpins our motivation for integrating metabolic profiling into this research, which we hypothesized to be able to offer novel insights into the metabolic pathways influencing exercise intolerance beyond what traditional metrics can capture (Figure 3).

### Metabolite Differences in PAH vs rTOF

Our study examined circulating metabolites in subjects with rTOF and PAH. Although both rTOF and PAH patients shared many similar metabolite signatures compared with control subjects, significant differences between the groups exist. Firstly, long-chain acylcarnitines (LCACs) (acylcarnitines with ≥14 carbon atoms) were significantly higher in PAH, compared with rTOF and control subjects. Next, the BCAAs (leucine, isoleucine, and valine) were higher in rTOF vs PAH patients, but were otherwise similar between PAH and control subjects. Finally, we observed significantly higher levels of one-carbon metabolites—dimethylglycine, choline, and guanidinoacetate—in PAH compared with rTOF patients. We also examined associations between serum metabolites and RV function in both rTOF and PAH. Our main findings in rTOF were inverse correlations between BCAA and RV functional parameters TAPSE and RVGLS. We observed that rTOF patients with lower valine had better RV function in terms of TAPSE and RVGLS. By contrast, there were no associations between valine and RV CMR parameters in PAH. In PAH, dimethylglycine associated positively with RV volume and RV mass, and negatively associated with RV function (RVEF, TAPSE, and RVGLS).

Among the numerous circulating metabolites that have been associated with an enhanced risk of cardiovascular disease,^18^ one of the most implicated group of metabolites is LCACs.^28^ The LCACs are derived from intermediates of fatty acid oxidation pathways. Accumulation of LCACs is likely a result of impaired mitochondrial oxidation of fatty acid fuel. High LCAC levels have been shown to be associated with key cardiovascular diseases, including HF, coronary artery disease, and cardiac arrhythmias.^29^, ^30^, ^31^, ^32^ Moreover, the LCAC–cardiovascular disease association has been reported to be proportional to disease severity in many studies,^33^^,^^34^ and Ahmad et al^34^ has demonstrated that LCACs were independently associated with adverse clinical outcomes in chronic HF patients. In our study, subjects from rTOF and PAH groups had increased levels of LCAC compared with control subjects, with more elevation in PAH, which is in line with previously reported studies^11^^,^^35^, ^36^, ^37^, ^38^ suggestive of myocardial mitochondrial dysfunction in both patient groups. We found that PAH patients had increased C18:1 and C18:2 compared with rTOF, associated with more dilated and hypertrophied RV, as well as higher RVEDV/LVEDV ratio (Figure 2). These observations may imply greater metabolomic and heart function changes in PAH patients, consistent with reductions in exercise capacity and in line with prior studies that have linked acylcarnitines to aerobic capacity.^39^

The circulating plasma BCAAs, particularly leucine, have potent nutrient signaling activity in cells that promote protein synthesis, cellular metabolism, and cell growth in a mammalian target of rapamycin (mTOR)-dependent manner. Increased levels of BCAAs and related metabolites are now widely considered to be a metabolomic hallmark of obesity, insulin resistance, and type 2 diabetes mellitus,^40^ and have been proposed to contribute to cardiovascular diseases by exacerbating systemic chronic inflammation, obesity, aging, diabetes, and other cardiovascular disease risk factors.^41^ In both patients and preclinical models, BCAAs play a key role in heart disease including HF.^18^ In a genetic mouse model, defective BCAA catabolism is linked to HF development,^42^ suggesting that elevated circulating BCAA seen in HF is linked to reduced tissue clearance. In HF with reduced ejection fraction patients (>1,000, single-center), leucine, valine, and BCAA-derived acylcarnitines as components of a plasma metabolite profile were observed to predict worse survival, independent of established HF with reduced ejection fraction risk prediction tools and biomarkers.^43^ By contrast, in some other populations (such as African Americans without cardiovascular disease and frail elderly individuals), high plasma BCAA concentrations predicted improved cardiovascular outcomes.^44^ Such findings appear contradictory, suggesting that increased plasma BCAA concentrations might be beneficial in specific populations and conditions.

In the current study, BCAA levels were comparable between PAH and control, while increased in rTOF. Previous studies have shown accumulation of BCAAs is linked to contractile dysfunction and adverse remodeling in failing hearts.^45^ We observed similar results for contractile dysfunction as demonstrated by the negative associations between BCAAs, TAPSE, and RVGLS in rTOF (Figure 2). BCAA metabolites play important roles in activating mTOR and promoting hypertrophy and fibrosis and are also important substrates for mitochondrial fuel metabolism. The differences observed between BCAA and response in PAH vs rTOF may represent differential roles played by mTOR signaling and mitochondrial fuel oxidation in these 2 conditions. Our result found that increasing BCAAs were associated with better RV remodeling (RVEDV/LVEDV), and we postulate that increased BCAAs may actively drive RV maladaptation in volume-overloaded rTOF as RV is dilated but not yet hypertrophied. As there are no available studies investigating the circulating plasma BCAAs in rTOF, the clinical implications of these findings remain unclear, and future studies are warranted. Given the importance of BCAA metabolic pathways in other forms of cardiovascular diseases, the differences noted in BCAA levels between rTOF and PAH subjects may be helpful in understanding how BCAAs contribute to myocardial dysfunction and remodeling under different loading conditions.

The one-carbon pathway is an important source of carbon for biosynthetic pathways such as nucleotide pathways as well as defending against oxidative stress and epigenetic modulation of genes. Our results highlighted increased accumulation of alanine and glutamine in both patient groups, which are both important mitochondrial fuel substrates. SAH level reflects the cell’s methylation capacity and its impairment may influence endothelial dysfunction, hypertrophy, fibrosis, and pulmonary vascular remodeling. In PAH, significant elevation of SAH was observed (Figure 1), and it was associated with RV dysfunction (Figure 2). In a recent study with 1,553 coronary artery disease patients,^46^ the investigators found that higher SAH levels were associated with increased all-cause and cardiovascular mortality, and each 1 SD increase in SAH raised cardiovascular death risk by 29%. This suggests SAH is a strong systemic marker of vascular risk and has potential for monitor PAH prognostication. In the body, dimethylglycine is produced in the one-carbon transfer cycle from choline via betaine in an enzyme-controlled transmethylation reaction. Studies have shown the importance of plasma dimethylglycine with its elevation as a risk marker of mortality in patients with coronary heart disease.^47^ The related methylated nitrogen compound trimethylamine oxide is now well-established as a marker for cardiovascular risk.^48^ The role of these one carbon-adjacent metabolites in PAH has not yet been well characterized in published literature. Indeed, the role of one-carbon metabolites in cardiovascular disease is still unclear as a largescale clinical trial to restore one-carbon balance did not reduce the risk of cardiovascular events.^49^ How perturbations in OCM are mechanistically linked to heart dysfunction is not known although studies suggest links to DNA methylation^50^ and mitochondrial function.^51^ In the current study, we found that significant increases in dimethylglycine was associated with RV adverse remodeling and RV dysfunction. Increased levels of SAH and dimethylglycine in PAH patients with corresponding relationships with RV dysfunction are hypothesis generating for a possible role of OCM in pressure overloaded hearts.

### Clinical Implications

In the literature, metabolomics analyses have been largely performed in HF cohorts with left heart dysfunction. Data from patients with predominantly RV dysfunction are scarce. Our advanced CMR methodology provided detailed evaluation of RV structure and function, which permitted a comprehensive evaluation of the relationship between diseases that implicate right heart function, paired against exercise capacity, with correlations with circulating plasma metabolite, that represent various loading conditions to the RV. Although they all have impaired RV function, higher RVEDV/LVEDV ratio, and exercise intolerance, the specific metabolite changes were distinct and correlations with RV CMR parameters and PVo_2_ were also different for individual metabolites. Additional ROC analysis has demonstrated the discriminative utility of individual metabolites compared with RVEF for predicting exercise intolerance in rTOF and PAH. A recent publication reviewed pharmacological manipulation of BCAA catabolism and dietary manipulation of BCAA intake in patients with cardiometabolic disease.^52^ Our study observations are preliminary, but they are important for paving the way to studies on metabolic modulators or interventions for BCAA metabolism in RV pressure- and volume-overload states. Our data, in conjunction with recent work in metabolomics that serve to prognosticate outcomes among PAH patients,^11^ support the emerging role of circulating metabolites, in tandem with deep imaging-based phenotyping,^10^ and CPET, to better understand their associations under different loading conditions.

### Study Limitations

The sample size in each subgroup was relatively small as we specified only subjects who had CMR, CPET, and metabolite profiling in the final analysis. Despite the small sample size, we observed significant associations between the disease groups. Future studies involving larger sample of patients, and cross validation of our findings, would be useful. The low number of clinical outcomes in our study cohort, comprising only 10 of 57 deaths (17.5%) in the PAH cohort, was insufficient to prognosticate these observations, although future work would be insightful. In terms of serum sampling, overnight fasting was not prespecified. Although nonfasting serum samples may generate analytic differences due to diet, prior studies suggest that variability in the measurements of most metabolites may not be significant with or without fasting.^53^ Lastly, we used rTOF as representative of RV volume overloading state, and such representation has been adopted in previous studies.^7^^,^^27^^,^^54^ Nevertheless, rTOF is a specific condition, and our findings may not be extrapolated to other RV volume overload states such as atrial septal defect or tricuspid insufficiency.

## Conclusions

Our study investigated RV function and remodeling by CMR, exercise by CPET, and plasma circulating metabolites in pressure-overloaded PAH and volume-overloaded rTOF, and explored the potential associations among them under different loading conditions. These exploratory findings may provide understandings of the interactions between heart, cardiopulmonary and circulating blood in PAH and rTOF, offering valuable insights into the management and future directions in respective patient cohort.

## Funding Support and Author Disclosures

This work was supported by the National Medical Research Council of Singapore (NMRC/OFIRG/0018/2016, MOH-000358, MOH-000351, MOH-001903-01, MOH-001200). The authors have reported that they have no relationships relevant to the contents of this paper to disclose.
