# Supplementary material for: Associations Between Right Ventricular Remodeling, Exercise and Circulating Metabolites in Volume and Pressure Overload States
Source: JACC Asia. 2025 Oct 29;6(2):210–24. doi: 10.1016/j.jacasi.2025.09.010 (PMC12926323; doi:10.1016/j.jacasi.2025.09.010)
Supplement: Supplementary Material [file mmc1.docx]

**Extended Methods**

**Study population**

The criteria for inclusion and exclusion are as follows:

Inclusion criteria

- For rTOF: 1) survivors of TOF repair more than one year after repair without residual intracardiac shunting, pulmonary stenosis (Doppler gradient>30mmHg), supraventricular arrhythmias, and significant ventricular arrhythmias (low grade>2); 2) aged: 8-80 years old.
- For PAH: 1) patients with clinically suspected or known primary PH belonging to one of the following subgroups of the Updated Dana Point Clinical Classification Group (idiopathic, heritable, drug or toxin induced or associated with one of connective tissue disease and congenital heart disease; 2) aged: 8-80 years old. For controls: 1) aged 12-80 years old; 2) asymptomatic and ambulant; and 3) resting blood pressure <140/90 mmHg.

The exclusion criteria for all subjects were: 1) contraindication to CMR examination; 2) non-cardiac illness with life expectancy of less than 2 years; 3) previous heart, kidney, liver or lung transplantation; and 4) pregnancy. Additional exclusion criteria for controls were 1) history of any major medical problems, cardiovascular disease or cardiovascular risk factor (e.g., hypertension, diabetes or dyslipidemia) or significant renal or lung disease; 2) concurrent use of medications for cardiovascular disease or cardiovascular risk factor management (e.g., for hypertension) and 3) heavy smoking (over 5 sticks per day or who had quit smoking for less than 12 months and had smoked over 5 sticks per day previously).

Patient groups had the following medical history: diabetes (rTOF: 4 (6.0%), PAH: 5 (8.8%)), hypertension (rTOF: 4 (6.0%), PAH: 9 (15.8%)), hyperlipidemia (rTOF: 3 (4.5%), PAH: 6 (10.5%)), and current smokers (rTOF: 7 (10.4%), PAH: 3 (4.8%)). There were four subtypes of PAH: 28 (49.1%) idiopathic PAH, 5 (8.8%) heritable PAH, 17 (29.8%), PAH associated with connective tissue disease and 7 (12.3%) PAH associated with congenital heart disease.

**Cardiopulmonary exercise test**

All adult subjects underwent exercise testing to maximal volition, on a Lode BV Corival electronically braked cycle ergometer (Groningen, Netherlands) within one week of the CMR scan. A ramp protocol was adapted for each subject. Minute ventilation (VE), oxygen consumption (VO_2_) and carbon dioxide output (VCO_2_) were acquired breath-by-breath and averaged over ten-second intervals. One metabolic equivalent (MET) was defined as the amount of oxygen consumed while sitting at rest (i.e., 3.5 ml of oxygen per kilogram body weight per minute). Peak oxygen uptake (PVO_2_) was the highest 10-second averaged sample obtained during exercise. % predicted PVO_2_ was calculated based on proposed normative values.^1^ VE/VCO_2_ slope was calculated via least squares linear regression (y = mx + b, m = slope) using VE and VCO_2_ values acquired from the start of exercise to peak.

**Metabolomics Profiling.** For acyl-carnitine analysis, thawed serum samples (50 μl) were spiked with 10 μl deuterium-labelled acyl-carnitine mixture and diluted with 400 µl methanol. After centrifugation of the mixture at 13,000 rpm for 5 min at 4 °C, the supernatant fraction was collected (100 μl) for acylcarnitine analysis. The solution was derivatized with 3M hydrochloric acid in methanol (Sigma Aldrich) and all compounds were ionized in positive mode using electrospray ionization. Acylcarnitine samples were run on an Agilent 6430 Triple Quadrupole LC/MS system (Agilent Technologies, CA, USA) with 80% methanol at flow rate of 0.4ml/min. The chromatograms were integrated using MassHunter Workstation Quantitative Analysis v10.0 (Agilent Technologies, CA, USA).

For analysis of nitrogen-containing compounds, 30 μL of sample was spiked with a mixture of deuterium-/carbon 13-labeled amino acids, then derivatized using 100 μL of 5% phenyl isothiocyanate. The mixture was reconstituted in 300 μL of 5 mM ammonium acetate in methanol, then diluted 1:4 using water. The compounds were separated and analyzed on a Waters Acquity UPLC BEH C18 column (1.7 µm, 2.1x50mm) using a Waters Acquity I-Class liquid chromatography system coupled to a Waters Xevo TQ-XS mass spectrometer (Waters Corp, Milford, MA, USA). The LC run was performed using 0.2% formic acid in water as mobile phase A and 0.2% formic acid in acetonitrile as mobile phase B, starting with initial gradient of 5% B and then increasing to 12% B at 1.5 min, 17.5% B at 2.7 min, 50% B at 4 min, 100% B at 4.5-5.0 min, before returning to 5% B from 5.0-5.8 min. The flow rate was held at 0.8 mL/min except during 4.7-5.1 min where it was raised to 1.0 mL/min. The column temperature was set at 50 °C and the injection volume was 5 µL. All compounds were ionized in positive mode using electrospray ionization. LC-MS/MS data was processed using Waters TargetLynx software v4.2 (Waters Corp, Milford, MA, USA).

A pooled quality control (QC) sample was prepared by mixing equal amounts (10 μl) of each extracted serum sample, then run regularly throughout the entire batch of both acyl-carnitine and nitrogen compound assays. Data from the pooled QC sample was used for determining assay precision and for batch normalization purposes.

**Reference**

1. Hansen JE, Sue DY, Wasserman K. Predicted values for clinical exercise testing. *Am Rev Respir Dis.* 1984;129:S49-55.

**Supplemental Table 1.** Acquisition parameters of cine imaging in two centers.

| Vendor | Philips | Siemens |
| --- | --- | --- |
| Magnetic field strength | 3.0T | 1.5T |
| Pulse sequence | Spoiled gradient echo | Spoiled gradient echo |
| TR/TE (ms) | 2.8/1.4 | 3.4/1.3 |
| Flip angle (°) | 45 | 72 |
| Field of view (mm^2^) | 320 x 320 | 320 x 260 |
| Slice thickness, mm | 8 | 8 |
| Cardiac phases | 30 | 30 |

*CMR* cardiovascular magnetic resonance, *TR* repetition time, *TE* echo time.

**Supplemental Table 2.** List of measured metabolites.

| **Acyl carnitine metabolites** | **Amino acid metabolites** | **Nitrogen pathway metabolites** |
| --- | --- | --- |
| C2 | Glycine | Dimethylglycine |
| C3 | Serine | Betaine |
| C4 | Threonine | β-Alanine |
| C5:1 | Alanine | Aspartic acid |
| C5 | Aspartic acid | 1-Methylhistidine |
| C4-OH | Asparagine | 3-Methylhistidine |
| C6 | Glutamic acid | Lysine |
| C5-OH/C3-DC | Glutamine | Kynurenine |
| C4-DC,C6-OH | Histidine | S-adenosylhomocysteine |
| C8:1 | Phenylalanine | Cysteine |
| C8 | Tyrosine | Taurine |
| C5-DC | Tryptophan | Homoarginine |
| C8:1-OH/C6:1-DC | Leucine | Symmetric dimethylarginine |
| C8-OH/C6-DC | Isoleucine | Asymmetric dimethylarginine |
| C10:3 | Valine | Guanidinoacetate |
| C10:2 | Methionine | Creatine |
| C10:1 | Arginine | Creatinine |
| C10 | Ornithine | Glutathione, reduced |
| C7-DC | Citrulline | Inosine |
| C8:1-DC | Proline | Hypoxanthine |
| C8-DC | Choline | Xanthine |
| C12:2 |  | Trimethylamine N-oxide |
| C12:1 |  | α-Aminobutyric acid |
| C12 |  | α-Aminoadipic acid |
| C12:2-OH/C10:2-DC |  | 5-Aminovaleric acid |
| C12:1-OH |  | Dihydroxyphenylalanine |
| C12-OH/C10-DC |  | Homocysteine |
| C14:3 |  | Methionine sulfoxide |
| C14:2 |  | Proline betaine |
| C14:1 |  | Tryptophan betaine |
| C14 |  | γ-Aminobutyric acid |
| C14:3-OH/C12:3-DC |  | Putrescine |
| C14:2-OH |  | Serotonin |
| C14:1-OH |  | Spermidine |
| C14-OH/C12-DC |  | Spermine |
| C16:3 |  | 3-Indoleacetic acid |
| C16:2 |  | Indole |
| C16:1 |  | 3-Indolepropionic acid |
| C16 |  | Cortisol |
| C16:3-OH/C14:3-DC |  | Cortisone |
| C16:2-OH |  | Trigonelline |
| C16:1-OH/C14:1-DC |  |  |
| C16-OH |  |  |
| C18:3 |  |  |
| C18:2 |  |  |
| C18:1 |  |  |
| C18 |  |  |
| C18:3-OH/C16:3-DC |  |  |
| C18:2-OH/C16:2-DC |  |  |
| C18:1-OH/C16:1-DC |  |  |
| C18-OH/C16-DC |  |  |
| C20:4 |  |  |
| C20:3 |  |  |
| C20:2 |  |  |
| C20:1 |  |  |
| C20 |  |  |
| C20:3-OH/C18:3-DC |  |  |
| C20:2-OH/C18:2-DC |  |  |
| C20:1-OH/C18:1-DC |  |  |
| C20-OH/C18-DC |  |  |
| C22:5 |  |  |
| C22:4 |  |  |
| C22:3 |  |  |
| C22:2 |  |  |
| C22:1 |  |  |
| C22 |  |  |
| C24 |  |  |
| C26 |  |  |
| C28 |  |  |

**Supplemental Table 3.** Factors (all the metabolites - nitrogen, AA and AC) identified by sparse principal component analysis and the associated individual components, description and variance.

| Factors | Description | Components | Proportion of variance accounted |
| --- | --- | --- | --- |
| 1 | Acyl-carnitines | C2, C4-OH, C6, C8, C8-OH/C6-DC, C10:1, C10, C8:1-DC, C8-DC, C12:2, C12:1, C12, C12-OH/C10-DC, C14:3, C14:2, C14:1, C14, C14:2-OH, C14:1-OH, C14-OH/C12-DC, C16:3, C16:2, C16:1, C16, C16:2-OH, C16:1-OH/C14:1-DC, C18:1, C18:1-OH/C16:1-DC, C18-OH/C16-DC | 0.140 |
| 2 | Amino acid-related | C3, C5, Glycine, Serine, Threonine, Alanine, Asparagine, Glutamic acid, Glutamine, Histidine, Phenylalanine, Tyrosine, Tryptophan, Leucine, Isoleucine, Valine, Methionine, Arginine, Ornithine, Citrulline, Proline, Choline, Dimethylglycine, β-Alanine, Aspartic Acid, 1-Methylhistidine, Lysine, Kynurenine, S-adenosylhomocysteine, Cysteine, Homoarginine, α-Aminobutyric acid, α-Aminoadipic acid, 5-Aminovaleric acid, Dihydroxyphenylalanine, Homocysteine, Indole | 0.098 |
| 3 | Acyl-carnitines and methylamine-related | C4, C5:1, C5, C5-OH/C3-DC, C4-DC/C6-OH, C5-DC, C10:2, C7-DC, C8:1-DC, C16:3-OH/C14:3-DC, C18, C18:3-OH/C16:3-DC, C18:2-OH/C16:2-DC, C18-OH/C16-DC, C20:4, C20:1, C20, C20:3-OH/C18:3-DC, C20:2-OH/C18:2-DC, C20:1-OH/C18:1-DC, C22:5, C22:2, C22:1, C22, C24, C26, C28, Citrulline, Choline, Dimethylglycine, Betaine, S-adenosylhomocysteine, Symmetric dimethylarginine, Creatinine, Inosine, Trimethylamine N-oxide, Proline betaine, Putrescine, Serotonin, Spermidine, Spermine, Trigonelline | 0.056 |
| 4 | Short- and medium chain acyl carnitines, amino acids and nitrogen transfer | C5:1, C5-OH/C3-DC, C4-DC/C6-OH, C8:1, C10:3, C7-DC, C12:2-OH/C10:2-DC, C16:3-OH/C14:3-DC, C18:2, C18:1, C20:3, C22:1, Glycine, Threonine, Aspartic acid, Glutamic acid, Phenylalanine, Leucine, Isoleucine, Methionine, Arginine, Ornithine, Citrulline, Proline, Dimethylglycine, β-Alanine, Aspartic Acid, Lysine, Kynurenine, S-adenosylhomocysteine, Taurine, Homoarginine, Guanidinoacetate, Creatine, Creatinine, Glutathione reduced, Inosine, Hypoxanthine, α-Aminobutyric acid, 5-Aminovaleric acid, Dihydroxyphenylalanine, Homocysteine, Methionine sulfoxide, Tryptophan betaine, γ-Aminobutyric acid, Putrescine, 3-Indoleacetic acid, 3-Indolepropionic acid, Cortisol, Cortisone | 0.053 |
| 5 | Acyl-carnitines and nitrogen transfer | C6, C5-OH/C3-DC, C10:2, C10:1, C10, C7-DC, C8-DC, C14, C14:2-OH, C16:1, C16, C16:3-OH/C14:3-DC, C16-OH, C18:3, C18:2, C18:1, C18, C18-OH/C16-DC, C20:4, C20:3, C20:2, C20:1, C20, C20:3-OH/C18:3-DC, C20:1-OH/C18:1-DC, C20-OH/C18-DC, C22:5, C22:4, C22:3, C26, Glycine, Serine, Threonine, Aspartic acid, Tryptophan, Arginine, Choline, Dimethylglycine, β-Alanine, Aspartic Acid, 1-Methylhistidine, 3-Methylhistidine, Kynurenine, Taurine, Symmetric dimethylarginine, Asymmetric dimethylarginine, Creatinine, Inosine, Hypoxanthine, α-Aminoadipic acid, 5-Aminovaleric acid, Dihydroxyphenylalanine, Homocysteine, γ-Aminobutyric acid, Serotonin | 0.059 |
| 6 | Short- and long-chain acyl-carnitines and nitrogen- and one-carbon transfer | C4-DC/C6-OH, C8:1, C5-DC, C8:1-OH/C6:1-DC, C8-OH/C6-DC, C10:3, C12:1-OH, C16:1-OH/C14:1-DC, C18, C18:2-OH/C16:2-DC, C20:4, C20:2, C20:1, C20, C22:5, C22:3, C22:1, C22, C24, C28, Serine, Glutamine, Phenylalanine, Ornithine, Choline, Dimethylglycine, β-Alanine, Aspartic Acid, Kynurenine, S-adenosylhomocysteine, Cysteine, Taurine, Symmetric dimethylarginine, Asymmetric dimethylarginine, Guanidinoacetate, Creatine, Creatinine, Inosine, Hypoxanthine, Xanthine, α-Aminoadipic acid, Dihydroxyphenylalanine, Homocysteine, Tryptophan betaine, Putrescine, Serotonin | 0.029 |
| 7 | Acyl-carnitines and nitrogen transfer | C3, C4, C5:1, C5, C4-DC/C6-OH, C8:1, C8:1-OH/C6:1-DC, C8-OH/C6-DC, C10:3, C10:2, C10, C7-DC, C8:1-DC, C12:2-OH/C10:2-DC, C12:1-OH, C12-OH/C10-DC, C14:3, C14:3-OH/C12:3-DC, C14:1-OH, C16, C18:2-OH/C16:2-DC, C20:1, C20:1-OH/C18:1-DC, C20-OH/C18-DC, C22:5, C22:2, C22:1, C28, Threonine, Alanine, Glutamine, 1-Methylhistidine, Homoarginine, Asymmetric dimethylarginine, Guanidinoacetate, Glutathione reduced, Trimethylamine N-oxide, α-Aminoadipic acid, Proline betaine, γ-Aminobutyric acid, Putrescine, Serotonin, Spermidine, Spermine | 0.036 |
| 8 | Short-chain acyl-carnitines and amino acids and one-carbon transfer | C3, C4, C5:1, C5, C5-OH/C3-DC, C4-DC/C6-OH, C5-DC, C12:2-OH/C10:2-DC, C18:3-OH/C16:3-DC, C18:2-OH/C16:2-DC, C20:1, C20, C20-OH/C18-DC, C22:1, C22, C24, Glycine, Serine, Threonine, Alanine, Asparagine, Glutamic acid, Glutamine, Leucine, Isoleucine, Valine, Arginine, Choline, Dimethylglycine, 3-Methylhistidine, S-adenosylhomocysteine, Cysteine, Asymmetric dimethylarginine, Creatinine, Hypoxanthine, Xanthine, α-Aminoadipic acid, 5-Aminovaleric acid, Methionine sulfoxide, Tryptophan betaine, Spermidine, Spermine | 0.026 |
| 9 | Acyl-carnitines and nitrogen Transfer | C3, C4-DC/C6-OH, C5-DC, C8-OH/C6-DC, C10:1, C12:2, C12:2-OH/C10:2-DC, C12:1-OH, C14:3-OH/C12:3-DC, C14:1-OH, C16:1, C16, C16:3-OH/C14:3-DC, C18:2, C20:3, C20:1, C20:1-OH/C18:1-DC, C20-OH/C18-DC, C22:5, C22:4, C22:1, C22, C24, C26, C28, Glycine, Threonine, Alanine, Ornithine, Citrulline, Proline, 1-Methylhistidine, Lysine, Homoarginine, Guanidinoacetate, Creatine, Creatinine, Trimethylamine N-oxide, α-Aminobutyric acid, Dihydroxyphenylalanine, Homocysteine, Methionine sulfoxide, Tryptophan betaine, γ-Aminobutyric acid, Putrescine, Serotonin, Spermidine, Spermine, Cortisone, Trigonelline | 0.018 |
| 10 | Acyl-carnitines and nitrogen transfer | C5, C6, C5-OH/C3-DC, C10:2, C7-DC, C8:1-DC, C12-OH/C10-DC, C14, C14:3-OH/C12:3-DC, C14:2-OH, C16, C16:3-OH/C14:3-DC, C16-OH, C18:3, C18, C18:3-OH/C16:3-DC, C18-OH/C16-DC, C20:4, C20:3, C20:2, C20, C20:1-OH/C18:1-DC, C22:5, C22:4, C22, C24, C26, Serine, Glutamine, Arginine, Ornithine, Choline, 1-Methylhistidine, 3-Methylhistidine, Lysine, Kynurenine, Taurine, Glutathione reduced, Inosine, Hypoxanthine, Xanthine, α-Aminobutyric acid, 5-Aminovaleric acid, Dihydroxyphenylalanine, Tryptophan betaine, Putrescine, Serotonin, 3-Indoleacetic acid, Indole, Cortisol, Trigonelline | 0.019 |

**Supplemental Table 4.** Individual metabolite means and comparisons between rTOF, PAH and controls

|  | **ANCOVA** | | **Pair-wise Comparisons from full model** | | | **Metabolite Factor Mean Values^#^** | | |
| --- | --- | --- | --- | --- | --- | --- | --- | --- |
| **Metabolite** | **Basic*** | **Fully Adjusted**** | **rTOF vs. Control** | **PAH vs. Control** | **PAH vs. rTOF** | **PAH**  **(n=57)** | **rTOF**  **(n=67)** | **Control (n=103)** |
| **Acyl-carnitine** | | | | | | | | |
| C16 | <0.0001 | <0.0001 | 0.004 | <0.0001 | 0.072 | 147.3 (9.5) | 131.9 (9.1) | 112.4 (10.3) |
| C18:1 | <0.0001 | <0.0001 | 0.081 | <0.0001 | 0.014 | 189.7 (14.9) | 159.2 (14.3) | 138.1 (16.2) |
| C14 | <0.0001 | <0.0001 | 0.007 | <0.0001 | 0.18 | 30.6 (2.9) | 26.8 (2.8) | 21.1 (3.1) |
| C18-OH/C16-DC | <0.0001 | <0.0001 | 0.20 | <0.0001 | 0.004 | 9.5 (0.8) | 7.6 (0.8) | 6.6 (0.9) |
| C18:2 | <0.0001 | 0.0002 | 0.090 | <0.0001 | 0.068 | 100.3 (9.0) | 85.6 (8.6) | 73.1 (9.8) |
| C4-OH | 0.036 | 0.67 |  |  |  | 34.9 (4.1) | 32.4 (3.9) | 32.7 (4.4) |
| C14-OH/C12-DC | 0.0001 | 0.021 | 0.48 | 0.017 | 0.37 | 10.2 (0.8) | 9.3 (0.8) | 8.5 (0.9) |
| **Branched-chain amino acids (BCAA) related** | | | | | | | | |
| C5-OH/C3-DC | 0.0007 | 0.080 |  |  |  | 31.3 (3.1) | 30.2 (3.0) | 26.8 (3.3) |
| C5-DC | 0.004 | 0.080 |  |  |  | 79.4 (12.6) | 62.7 (12.2) | 59.8 (13.8) |
| C3 | 0.30 | 0.75 |  |  |  | 528.1 (43.8) | 550.8 (42.1) | 536.0 (47.6) |
| C5 | 0.11 | 0.82 |  |  |  | 122.1 (13.0) | 123.6 (12.5) | 118.5 (14.1) |
| Valine | <0.0001 | 0.0001 | 0.001 | 1.0 | 0.001 | 331.2 (20.7) | 385.2 (19.9) | 335.5 (22.5) |
| Isoleucine | 0.017 | 0.032 | 0.14 | 1.0 | 0.047 | 107.0 (9.7) | 123.8 (9.3) | 111.5 (10.5) |
| Leucine | 0.016 | 0.025 | 0.21 | 0.90 | 0.026 | 158.2 (13.3) | 183.5 (12.8) | 168.1 (14.5) |
| **One-carbon metabolites** | | | | | | | | |
| Homocysteine | <0.0001 | <0.0001 | <0.0001 | <0.0001 | 1.0 | 0.04 (0.004) | 0.04 (0.003) | 0.03 (0.004) |
| Dimethylglycine | <0.0001 | <0.0001 | <0.0001 | <0.0001 | 0.013 | 0.006 (0.0004) | 0.005 (0.0004) | 0.004 (0.0004) |
| S-adenosylhomocysteine | 0.0003 | 0.011 | 0.63 | 0.008 | 0.17 | 0.009 (0.002) | 0.006 (0.002) | 0.005 (0.002) |
| Glutamine | <0.0001 | <0.0001 | <0.0001 | <0.0001 | 0.79 | 1059.3 (63.6) | 1110.4 (61.2) | 860.9 (69.2) |
| Glycine | 0.0005 | 0.0001 | <0.0001 | 0.001 | 1.0 | 322.6 (24.9) | 320.4 (24.0) | 259.1 (27.1) |
| Alanine | <0.0001 | <0.0001 | <0.0001 | 0.005 | 1.0 | 515.9 (31.2) | 531.5 (30.0) | 444.3 (34.0) |
| Serine | 0.0019 | 0.0073 | 0.017 | 0.030 | 1.0 | 177.0 (20.4) | 175.0 (19.7) | 138.9 (22.2) |
| Taurine | 0.015 | 0.060 |  |  |  | 0.25 (0.07) | 0.32 (0.07) | 0.22 (0.07) |
| Choline | <0.0001 | <0.0001 | 0.83 | <0.0001 | 0.001 | 11.6 (0.9) | 9.3 (0.9) | 8.7 (1.0) |
| Methionine | 0.28 | 0.42 |  |  |  | 33.7 (3.1) | 36.5 (2.9) | 34.6 (3.3) |
| Creatine | 0.12 | 0.30 |  |  |  | 0.10 (0.02) | 0.11 (0.02) | 0.13 (0.02) |
| Guanidinoacetate | 0.0025 | 0.021 | 1.0 | 0.051 | 0.030 | 0.0027 (0.0003) | 0.0034 (0.0003) | 0.0033 (0.0003) |

**^#^**Data are represented as least square means, adjusted for 10 covariates (age, sex, body surface area, systolic and diastolic blood pressure, heart rate, diabetes, hyperlipidemia, hypertension and current smoker). SEM is provided beneath each value.

* *P* values for basic model, adjusted for age and sex

** *P* values for full model, adjusted for age, sex, body surface area, systolic and diastolic blood pressure, heart rate, diabetes, hyperlipidemia, hypertension and current smoker.


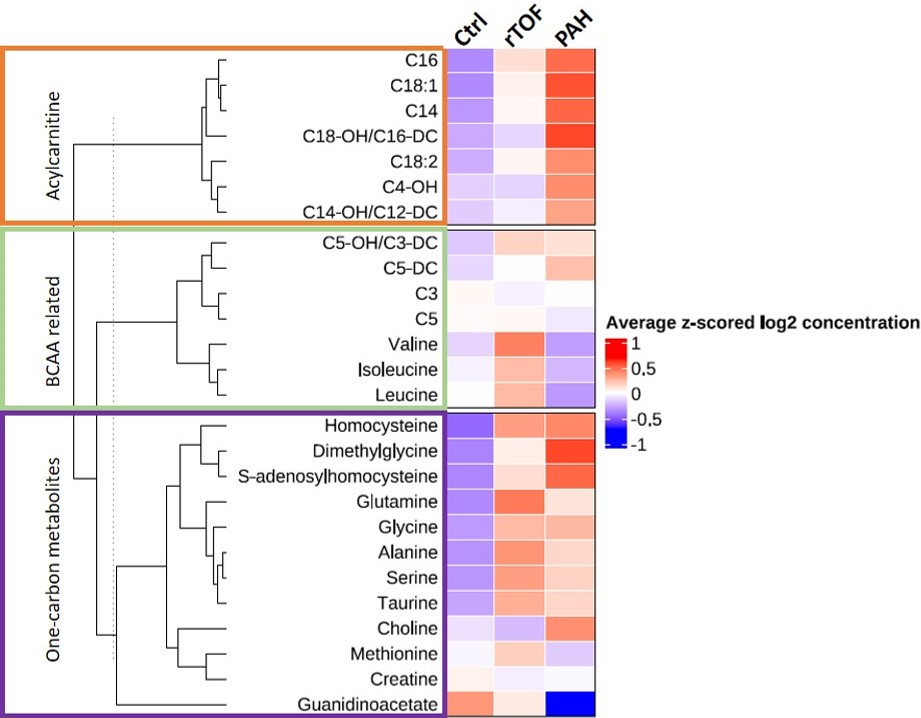


**Supplemental Figure 1.** Heat map for the individual metabolites selected in Table S3 categorized into 3 categories: acyl-carnitine, branched-chain amino acids (BCAA), one-carbon metabolism. All the metabolites were normalized by log2 transformation, standardized by the z-score and averaged.
